# Quantitative bias analyses to address measurement error in time-to-event endpoints

**DOI:** 10.1093/aje/kwag027

**Published:** 2026-02-06

**Authors:** Benjamin Ackerman, Ryan W Gan, Youyi Zhang, Jennifer Hayden, Jocelyn R Wang, Craig S Meyer, Juned Siddique, Jennifer L Lund, Janick Weberpals, Sebastian Schneeweiss, Til Stürmer, James Roose, Omar Nadeem, Noopur Raje, Sikander Ailawadhi, Smith Giri, Laura Hester, Jason Brayer, Ashita S Batavia

**Affiliations:** Johnson & Johnson, Raritan, NJ, United States; Johnson & Johnson, Raritan, NJ, United States; Johnson & Johnson, Raritan, NJ, United States; Johnson & Johnson, Raritan, NJ, United States; Johnson & Johnson, Raritan, NJ, United States; Johnson & Johnson, Raritan, NJ, United States; Department of Preventive Medicine, Feinberg School of Medicine, Northwestern University, Chicago, IL, United States; Department of Epidemiology, University of North Carolina at Chapel Hill, Chapel Hill, NC, United States; Division of Pharmacoepidemiology and Pharmacoeconomics, Brigham and Women’s Hospital, Harvard Medical School, Boston, MA, United States; Division of Pharmacoepidemiology and Pharmacoeconomics, Brigham and Women’s Hospital, Harvard Medical School, Boston, MA, United States; Department of Epidemiology, University of North Carolina at Chapel Hill, Chapel Hill, NC, United States; Flatiron Health, New York, NY, United States; Department of Hematology and Oncology, Dana-Farber Cancer Institute, Harvard Medical School, Boston, MA, United States; Center for Multiple Myeloma, Massachusetts General Hospital Cancer Center, Boston, MA, United States; Department of Hematology, Mayo Clinic, Jacksonville, FL, United States; Division of Hematology and Oncology, Department of Medicine, University of Alabama at Birmingham, Birmingham, AL, United States; Johnson & Johnson, Raritan, NJ, United States; Johnson & Johnson, Raritan, NJ, United States; Johnson & Johnson, Raritan, NJ, United States

**Keywords:** measurement error, real-world data (RWD), oncology, external control arm, quantitative bias analysis, misclassification bias, surveillance bias, endpoints

## Abstract

When using real-world data to construct an external comparator arm for a single-arm trial, there may be differences in how and when patients are assessed for disease between trial and real-world settings. Such differences can generate outcome measurement error when comparing time-to-event endpoints and lead to biased findings. Recent methods have been developed to mitigate measurement error bias in real-world endpoints; however, they rely on the existence of a validation sample, ie, data on a set of patients where both the “true” trial-like and “mis-measured” real-world measures are collected. We demonstrate how novel statistical methods can be leveraged as quantitative bias analyses (QBA) to contextualize real-world evidence findings when outcome measurement error is of concern, but validation samples are infeasible to collect. Quantitative bias analyses allow researchers to set plausible ranges for the amount of error when not directly measurable. We highlight how to conduct QBA with two recent methods, cumulative incidence curve correction and survival regression calibration, and illustrate how to generate plausible parameter values through simulation. We provide an illustrative QBA example in a cohort of real-world patients with newly diagnosed multiple myeloma and provide practical guidance to apply QBA for outcome measurement error and interpret results.

## Introduction

There has been an increasing recognition of the value of real-world data (RWD) in constructing external comparator arms (ECAs) to contextualize efficacy findings from oncology and rare disease single-arm trials. While RWD can complement clinical trials, these studies may be subject to many types of systematic biases that limit their utility, namely due to differences between trial and real-world care settings.^1^ Recognizing that biases in real-world evidence (RWE) studies present significant challenges for interpretation of ECAs using RWD, the US Food and Drug Administration (FDA) has issued draft guidance^2^ on comparability challenges when conducting ECAs. Measurement error (or information bias) in study endpoints is one such source of bias that is highlighted in FDA guidance, along with other common sources like confounding and selection bias; however, outcome measurement error is often less studied or addressed in research. Measurement error of time-to-event (TTE) endpoints in ECA studies using RWD comparators is particularly challenging compared to binary endpoints due to differences in both *how* an event is classified (eg, misclassification at a particular assessment due to incorrect outcome status assignment) and *when* an event is recorded (eg, delays attributed to variable time between clinical assessments). In other words, the impact of the error can depend on both the accuracy of each assessment as well as the amount of time between each assessment. Failure to account for outcome measurement error in RWD relative to trial standards can lead to bias and result in misleading conclusions, particularly when this error is differential with respect to comparisons between groups, as could be the case in an externally controlled trial using RWD.

Methods to adjust for outcome measurement error tend to focus on either misclassification of a categorical outcome (incorrect assignment of one class to another) or measurement error of a continuous outcome; however, less research has focused to date on TTE outcomes, where both event *types* and event *times* may be error-prone.^3^^-^^5^ Two novel methods have been recently developed to address TTE outcome measurement error^6^^,^^7^; however, applying these methods requires a validation sample, defined as a study where both the true and mis-measured versions of the outcome are collected, to model the relationship between the two and calibrate mis-measured outcomes in the full study sample. In practice, validation samples may not be readily available (or feasible to design) in RWD, warranting further development of existing methods to conduct quantitative bias analyses (QBA) and illustrate how outcome measurement error could affect study results.

Quantitative bias analyses include tools that can contextualize systematic biases in RWD, allowing for quantification of the direction, magnitude, and uncertainty of these biases.^8^^-^^10^ These methods can range from addressing a single bias, such as unmeasured confounding or exposure measurement error, to more complex modeling approaches that handle multiple biases simultaneously. Despite their apparent utility and recommended adoption in regulatory guidance,^11^ there has been limited uptake of these approaches in studies of drug safety and effectiveness using RWD, in part due to limited guidance in identifying and assigning reasonable bias parameters to these statistical models, and gaps in available software for statistical modeling. Despite these limitations, QBA methods can be useful when analyzing RWD, allowing researchers to explicitly evaluate systematic biases into the modeling process rather than implicitly rationalizing how biases, like outcome measurement error, may influence study results.

In this paper, we demonstrate how TTE outcome measurement error correction methods can be adapted and applied as QBA for RWE without validation data. We provide guidance on how to set plausible ranges for these methods’ necessary bias parameters, leveraging simulation techniques, descriptive RWD analyses, and clinical expertise. Using RWD on patients with newly diagnosed multiple myeloma (NDMM), we apply these QBA techniques as an illustrative example and discuss how to interpret their output to contextualize the impact of measurement error on estimates of real-world median progression-free survival (PFS). Collectively, our findings support the incorporation of QBA methods into analyses of RWD, allowing for a more nuanced evaluation of evidence by explicitly accounting for outcome measurement error bias.

### Motivating example

**Figure 1:**
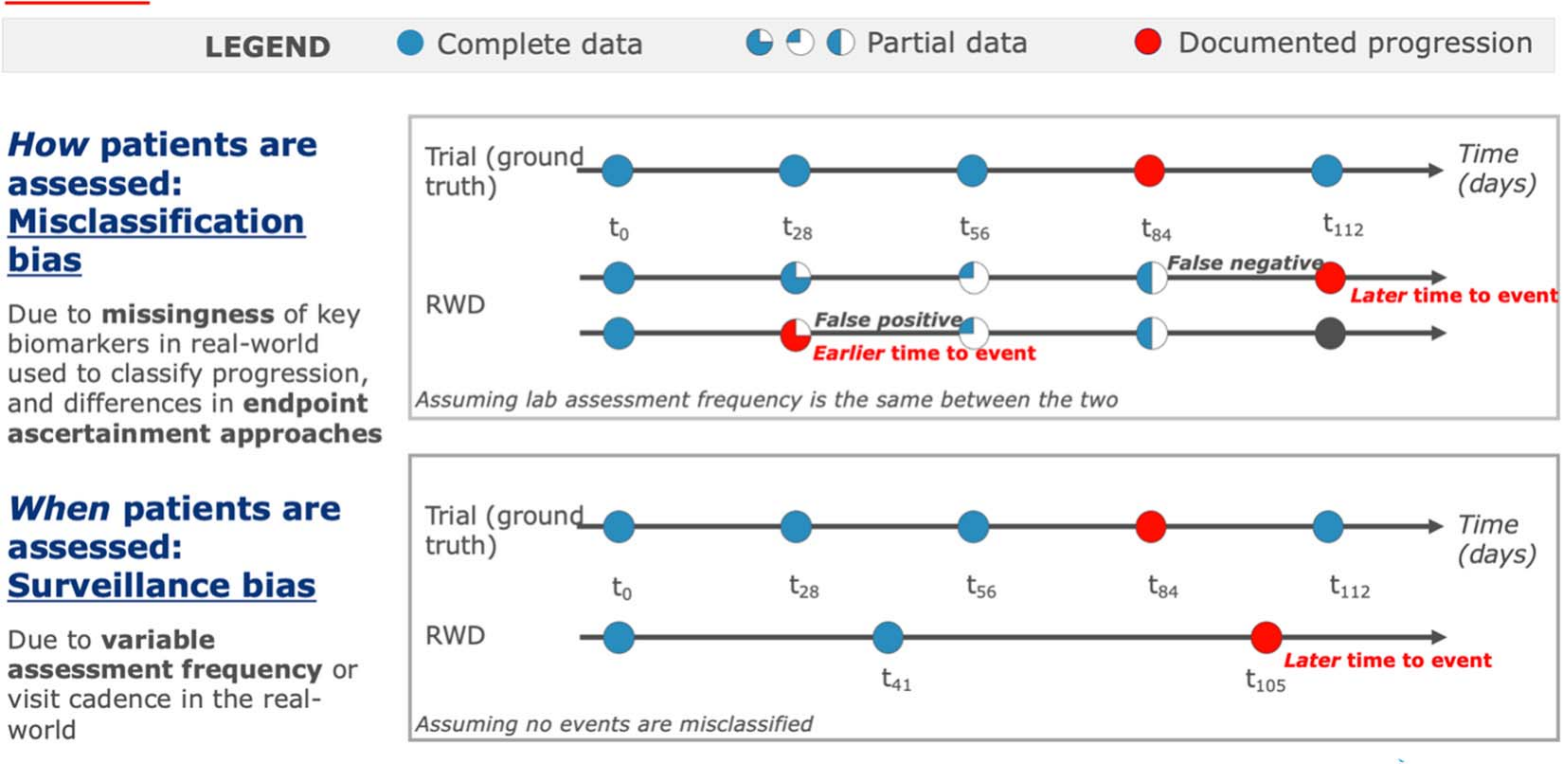
Illustration of assessment schedule bias and misclassification in RWD vs trial. Note that while the illustration of surveillance bias depicts delayed detection of an event relative to trial standards, it is also plausible that real-world assessment frequencies could also yield progression detection *earlier* than trial schedules. Abbreviation: RWD, real-world data.

Use of RWD as an ECA to contextualize clinical trial data is an example where outcome measurement error is a concern, and where QBA methods can be used to understand the potential impact of this bias. Consider a single-arm trial proposed to evaluate PFS for a new cancer therapeutic, where results may be contextualized by constructing an external comparator arm from RWD. Progression-free survival is a composite endpoint measuring the time from treatment start to either disease progression or death and is an important early surrogate endpoint for overall survival in oncology clinical trials. In multiple myeloma clinical trials, assessment of disease progression follows a protocol with a well-defined assessment schedule and standardized evaluation process following the International Myeloma Working Group (IMWG) criteria. In contrast to solid tumor cancers, where disease assessment standards are predominantly imaging-based,^12^ progression events in MM per IMWG criteria are determined by changes in serum (SPEP) and urine M-protein (UPEP), free-light chains (FLCs), bone marrow plasma cells, and presence of new bone lesions or plasmacytomas^13^ (see Table S1 for all abbreviations). In real-world care settings, myeloma patients generally do not follow a strict disease assessment schedule and are not assessed for progression using full IMWG criteria. Because of this, progression events captured during real-world care using real-world progression algorithms operationally defined to closely resemble the IMWG criteria^14^ may differ from progression events captured in clinical trials (Figure 1), resulting in bias due to measurement error. In other words, estimates of median real-world PFS (rwPFS) may be biased relative to the trial based on differences in how and when patients are assessed for progressive disease. We demonstrate throughout our illustrative example how TTE measurement error correction methods can be applied as QBA to adjust rwPFS estimates, contextualizing concerns based on endpoint measurement differences between trial and real-world settings.

## Methods

To address outcome measurement error in rwPFS, we can apply recently developed statistical methods: cumulative incidence curve correction (CICC) and survival regression calibration (SRC).^6^^,^^7^ Broadly, these methods entail 1) estimating the (“mis-measured”) survival curve in RWD of interest, 2) modeling the relationship between true (trial-like) and mis-measured (real-world) outcomes in a validation sample and summarizing their differences in bias parameters, and then 3) calibrating the real-world curve, shifting it “up” or “down” according to the estimated parameters. The methods highlight different ways to define and estimate bias parameters between trial and RWD endpoints; one estimates the error rates of the event detection, while the other estimates the bias *due* to the event detection errors (see Table 1). Note that both approaches are valuable and can provide complementary evidence.

**Table 1:** Overview of statistical methods for time-to-event outcome measurement error correction.

**Features of methods**	**CICC**	**SRC**
General concept	Shifted cumulative distribution function based on sensitivity, false positive rate, and delayed event detection rate	Shifted survival curve based on calibrated Weibull scale and shape parameters
Underlying survival model	Any nonparametric survival function estimation method	Parametric accelerated failure time Weibull model
Necessary input parameters	Sensitivity False positive rate Delayed event detection rate	Scale parameter Shape parameter
Identification of parameters	Estimation in a validation sample Approximation through simulation, or plausible ranges based on expert opinion (QBA)	
Method citation	Edwards et al. (2025) *Risk functions with outcome measurement error.* Biostatistics.^6^	Ackerman et al. (2025) *Regression calibration for time-to-event outcomes: Mitigating bias due to measurement error in real-world endpoints.* Epidemiologic Methods.^7^

Abbreviations: CICC, cumulative incidence curve correction; QBA, quantitative bias analyses; SRC, survival regression calibration.

When applying these methods in RWD, the data required to complete step 2 (ie, a “validation sample” where patients have both versions of the outcome collected) may not be readily available or feasible to curate. Therefore, we consider how to leverage existing methods for QBA, wherein we set *plausible ranges* for the bias parameters when we cannot directly estimate them.

### Leveraging methods as QBA when validation samples are not available

Identifying reasonable ranges for QBA bias parameters can be done by leveraging clinical expertise, study protocol details, and statistical simulation. Note there is limited published research and knowledge to date about the impact of measurement error biases on TTE outcomes^15–17^, and thus expert opinions may be more qualitative than quantitative. Statistical simulations can be used to translate clinical experts' input about factors that generate measurement error that contribute to the bias into the appropriate QBA parameters, particularly when the bias parameters themselves do not have intuitive value approximations. See Table 2 for an overview of how these simulation techniques can be used depending on the information available to the researcher. We now highlight an example of one such approach in an illustrative example.

**Table 2:** Guidance for using simulations to estimate bias parameters.

**Data available describing the “ground truth” trial conditions**	**How to generate “ground truth”**	**How to simulate misclassification bias**	**How to simulate surveillance bias**
Historical trial individual-level patient data	Use de-identified historical data available and observed outcomes for those patients	Impose missingness on key biomarkers, use real-world derivation approach to get mis-measured outcome	Mixture distribution → shift assessment and outcome times
Summary statistics describing population size and outcomes	Simulate outcomes according to parametric distributions using available summary statistics	Impose false positives and negatives of events based on rates from literature and subject matter expertise	

### Illustrative example

Suppose we want to answer the question: what would an estimate of median rwPFS be, had patients been assessed for disease progression according to trial-like standards? We illustrate an answer using RWD from the Flatiron Health Research Database (FHRD) from 2015 to 2023. The Flatiron Health Research Database is a longitudinal database, comprising de-identified patient-level structured and unstructured data, curated via technology-enabled abstraction. We obtain an unadjusted (ie, mismeasured) estimate of median rwPFS for a cohort of NDMM patients who received lenalidomide and dexamethasone as their first line of treatment. Since there is no “trial-like outcome” captured in the FHRD, and thus no validation sample is available for modeling, we apply TTE measurement error methods as QBA to shift the survival curve by plausible amounts, accounting for differences between real-world and trial outcome measurement approaches.

The statistical parameters required to apply CICC and SRC relate to our motivating example, at a high level, as follows: For CICC, the three parameters describe how likely a progression event is to be identified correctly, or not, at a given time with both real-world and IMWG criteria, along with the amount of time that elapses with delayed detections. For SRC, the two parameters together describe the amount which real-world PFS estimates may be shifted toward longer times due to delayed disease progression event detection and missed events, as quantified through Weibull regression. However, insights from Myeloma and RWD experts on values for these exact parameters may be more qualitative in nature, particularly given the limited published research on the topic. Therefore, simulations may help utilize knowledge or opinions on simpler, more intuitive parameters that describe the presence of measurement errors, and then calculate the corresponding bias parameters for QBA. We now describe how to determine those plausible errors and translate them into QBA parameters via simulation.

### Simulation to set plausible bias parameters and conduct QBA

Our goal is to set plausible ranges for the bias parameters to calibrate rwPFS using CICC and SRC such that it better reflects trial-like measurement standards. We simulate data, first defining the “ground truth,” or what we assume the PFS outcome distribution to be without measurement error. When a historical trial on a relevant population is available, such data can be leveraged to inform assumptions. Otherwise, summary statistics like median PFS, median overall survival, or Kaplan–Meier curves from published literature can be used to infer and simulate individual patient data.^18^ Here, “trial-like” PFS times are simulated from an exponential distribution with varying censoring rates and follow-up time per trial characteristics (see example code in Appendix S1). For our illustrative QBA, we simulate 365 patients using the published median PFS of 34.4 months from the control arm of the MAIA trial, which evaluated efficacy of daratumumab, lenalidomide, and dexamethasone vs lenalidomide and dexamethasone alone in NDMM patients.^19^ We then introduce real-world–like measurement error due to misclassified events and varying assessment frequencies, generating a “mis-measured” rwPFS version of the originally simulated outcome (details in next section). We utilize both the “trial-like” and “real-world-like” simulated versions of PFS to calculate each method’s QBA bias parameters, translating our measurement error assumptions. To estimate the bias parameters and report on their uncertainty, we simulate 1,000 random datasets, estimate the QBA bias parameters within each dataset, calibrate our original rwPFS estimate 1,000 times according to the parameter distributions, and construct simulation intervals based on the quantiles from the distributions of the simulated parameter estimates.

#### Simulating surveillance bias

Recall from Figure 1 that one contributing factor to measurement error in RWD is the difference in assessment schedules between a trial and real-world setting. Variability in time between disease assessments in routine clinical practice can lead to disease progression events being detected later in the real-world than they would have been in trial settings. For our illustrative example, we emulate and impose this bias by first characterizing the time between lab collections of SPEP and FLC in RWD, and see that the mode of time between assessments for patients is every 28 days, but the distribution is spread out with far more variability than a trial setting (see Figure S1). We approximate this by simulating mixture distributions of assessment times and then shifting the “true” patient progression times to adhere to the simulated disease assessment schedules, thereby generating surveillance bias (see example code in Appendix S1).

#### Simulating misclassification bias

Another contributing factor to measurement error bias is the misclassification of progression events. In MM RWD, only certain commonly collected biomarkers, like SPEP and FLC, are typically available to derive progression outcomes, rather than the full set required per IMWG criteria. This missingness, coupled with the use of more flexible real-world progression algorithms in lieu of full IMWG criteria, can lead to misclassification bias. In our simulation, we use information on how biomarker missingness and real-world algorithms yield misclassified events in the form of sensitivity and specificity to derive a “mis-measured” real-world version of the outcome where certain progression events are missed, and others are erroneously detected early. Furthermore if a disease progression event is missed (false negative), or if a progression event is captured earlier (false positive), we simulate an event detection delay or premature capture of approximately 1 to 2 months, respectively. Here, we leverage expert clinical opinion on the progression determination and assessment frequency differences between trial and routine clinical care settings to inform our parameters. More specifically, we presume that true progression events are more likely to be under-detected in RWD, and that premature false event detection is less likely. In accordance with this, we assume that 50% of patients whose PFS is truly defined by a progression event are misclassified, such that the progression event is detected in RWD 1-2 months later.^3^ We also assume that for 20% of patients who do not progress at any point in the study (ie, their PFS time is truly defined by death or censoring), a progression event is *falsely* detected 1-2 months earlier than their PFS time based on clinical expertise.

#### Calculating bias parameters and conducting QBA

After simulating misclassification and surveillance bias, the respective QBA parameters for CICC and SRC are calculated in the simulated datasets. The estimated rwPFS curve from the FHRD is then shifted according to the set of parameters per method from each dataset, generating a distribution of plausible PFS curves that account for the plausible amount of measurement error. For CICC, the three parameters are plugged in to the estimator of the cumulative incidence function described in Edwards et al.; for SRC, the two parameters are used to offset the Weibull parameters originally estimated in the FHRD according to Ackerman et al. We next present results of how these simulated data can be used for QBA in an illustrative example.

### Results

#### Simulated plausible measurement error parameter estimates

**Figure 2:**
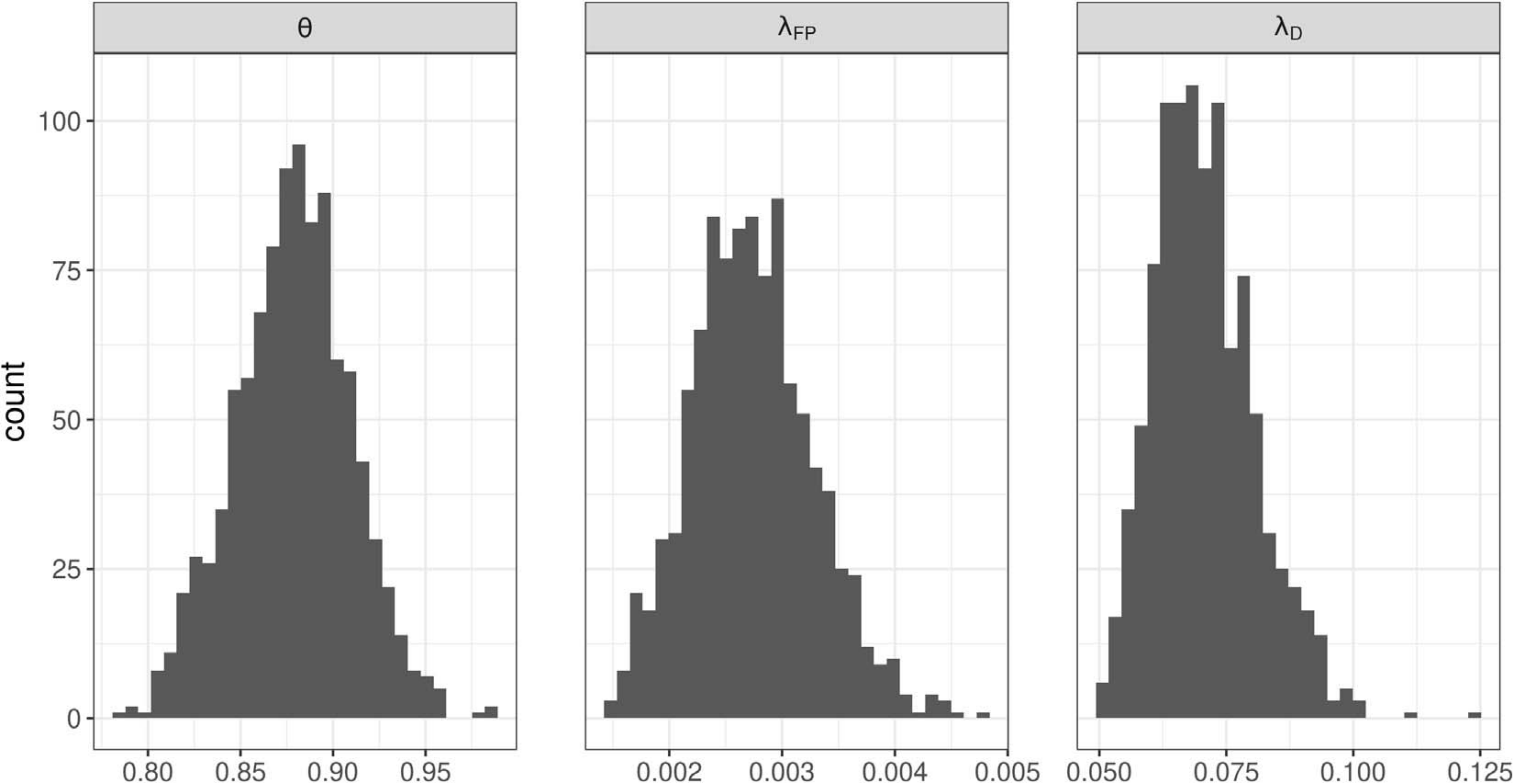
CICC QBA parameters estimated via simulation: *θ* (sensitivity parameter), λ_FP_ (false positive rate), and λ_D_ (late detection rate). Abbreviations: CICC, cumulative incidence curve correction; QBA, quantitative bias analyses.

**Figure 3:**
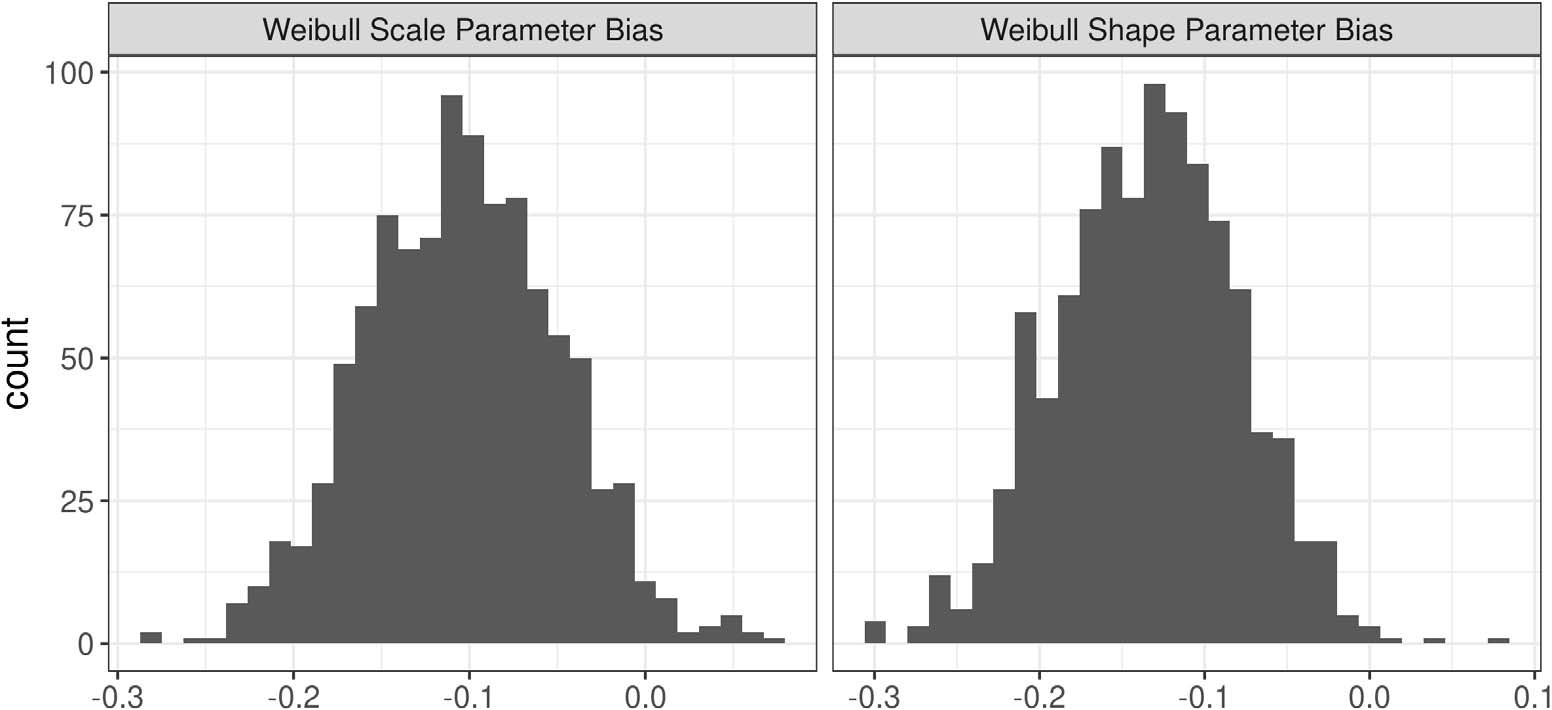
SRC QBA parameters estimated via simulation. Abbreviations: SRC, survival regression calibration; QBA, quantitative bias analyses.

We simulated PFS times both with and without measurement error for 365 patients. By comparing the simulated times, we estimate distributions of bias parameters for the two methods of interest, CICC (Figure 2) and SRC (Figure 3). These distributions describe the plausible amounts of measurement error that may bias our observed estimate of real-world PFS, which we then address via QBA.

For CICC, the “theta” parameter (*θ*) (Figure 2) represents the proportion of progression events under both real-world and trial scenarios. In this case, the mean theta of 0.83 suggests that 83% of trial progression events would also be captured under our real-world progression scenario, had a false positive event not already occurred. The mean false positive event rate parameter (Figure 2) (λ_FP_) of 0.0027 suggests that 2.7 real-world progression events would be incorrect for every 1000 months contributed by patients classified as progressing in our real-world scenario but who were not classified as progressing in our trial. This number is comparatively smaller, when compared with the late detection rate parameter (Figure 2) (λ_D_). This mean λ_D_ of 0.07 suggests that 7 real-world progression events are detected later in time for every 100 months of time contributed by patients classified as progressing in our trial. The simulated distributions for CICC lead to bias parameter values that align with clinical expectations, where most progression events in the real-world are captured within a reasonable time, and late detections occur at a higher rate compared to false positives.

For SRC, both the scale and the shape bias parameters are defined as the “ground truth” values minus the “mis-measured” values (Figure 3). The mean bias (−0.11, −0.014) for each respective simulated parameter distribution appear to be below zero, which reflects a translation of our chosen measurement error simulation parameters to an estimate of real-world PFS shifted toward *later* times than trial PFS. Note this is consistent with the interpretation and clinical expectation expressed for CICC.

#### QBA results: calibrating estimates of real-world PFS

**Figure 4:**
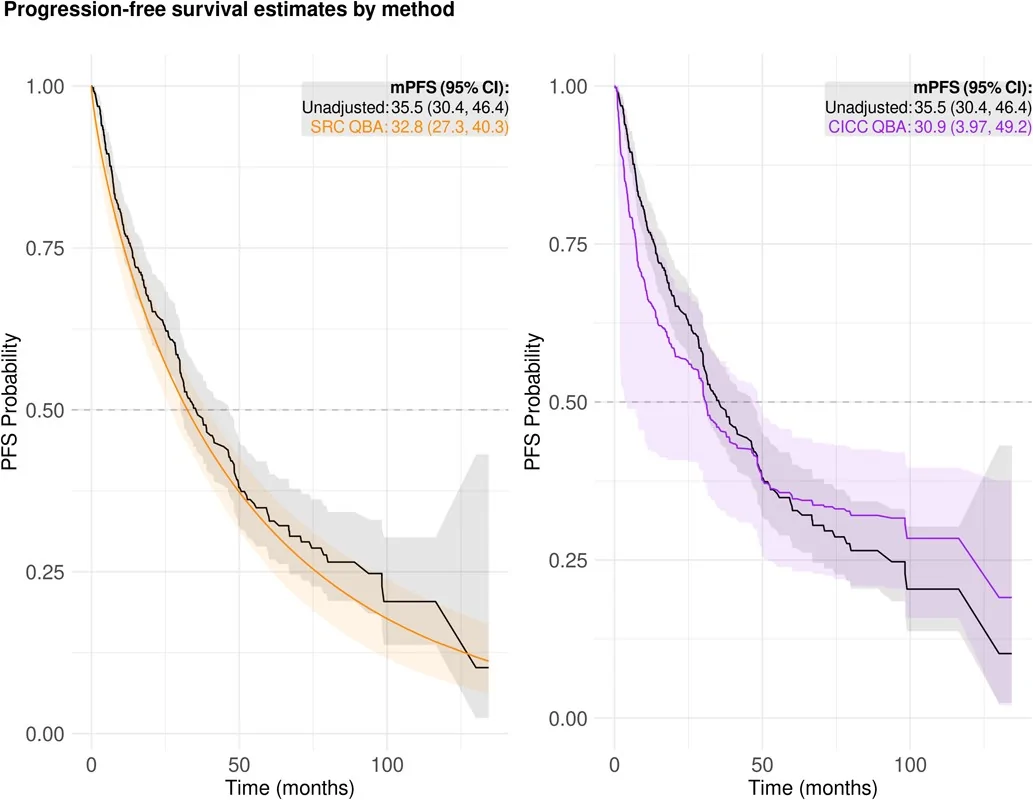
PFS curves unadjusted, and accounting for measurement error CICC or SRC. Abbreviations: CICC, cumulative incidence curve correction; PFS, progression-free survival; SRC, survival regression calibration.

Real-world PFS curves before and after application of QBA methods adjusting for TTE measurement error are presented in Figure 4. The unadjusted real-world median PFS (mPFS) was estimated as 35.5 months (95% CI, 30.4-46.4). After applying CICC as QBA using the bias parameters from Figure 2, mPFS on average shifted towards slightly shorter times (mPFS, 30.9 months; 95% CI, 3.97-49.2). In addition, the PFS curve shift was more pronounced for later times, as the late detection rate (λ_D_) had more influence than the false positive detection rate (λ_FP_). While mPFS appeared slightly shorter than the unadjusted analysis, the simulation intervals for the two estimates were unsurprisingly overlapping, as the QBA accounted for uncertainty in both the bias parameters and standard sampling error. This also aligns with clinical expectations, as a shift of 4-5 months in the mPFS point estimate may not indicate a clinically significant difference between PFS estimates in an NDMM patient cohort.

When applying SRC as QBA, the measurement error parameters in Figure 4 shifted the PFS curve estimate to be slightly worse than the unadjusted curve, resulting in a mPFS estimate of 32.8 months (95%CI, 27.3-40.3). Note the resulting curve was smooth, compared to the step function of the non-parametric Kaplan–Meier estimate, due to the Weibull parametric model. Again, while the median time appeared slightly shorter than the unadjusted analysis, the overlapping simulation intervals and minimal shift in the point estimate suggest minimal impact of the considered degree of measurement error on bias in this NDMM patient cohort.

## Discussion

We have presented how novel statistical methods can be leveraged as QBA to contextualize uncertainties in external comparators constructed using RWD due to TTE measurement error when validation samples are unavailable or infeasible. This work and methodology allow us to analyze RWD and make our implicit assumptions about measurement error in endpoints explicit in a quantitative way. For the ECA use-case, we assume trial endpoints are measured per a “gold standard” (ie, they are “error-free”), and real-world endpoints are mis-measured according to that same standard. It is plausible that trial endpoints *themselves* may also be prone to measurement error relative to a particular standard, which would warrant further extension of the methods presented in this paper.

Through the total evidence from our illustration of these QBA methods in an example analysis of NDMM RWD, we see that while measurement error may be present in a real-world endpoint, the plausible amounts we considered seem to have a minimal effect on bias in median real-world PFS estimates. This analysis provides valuable insights into the role of measurement error when estimating real-world PFS.

There are some differences to note in the application of CICC and SRC. Cumulative incidence curve correction quantifies the specific errors that are important contributions of bias such as false positive rate and late detection rate, which can inform how real-world PFS and trial PFS are different. Survival regression calibration focuses on shifting the survival function from a Weibull accelerated failure time model after estimation and is not focused, like CICC, on decomposing important information like false positive rate and late detection. In other words, whereas CICC estimates the *error* that generates bias, SRC estimates the *bias* itself. A benefit of SRC using parametric models is the ability to more readily incorporate methods to handle other TTE biases, such as left truncation and interval censoring for surveillance bias, when estimating the measurement error impact. Furthermore, SRC may produce survival estimates that are less variable and sensitive than CICC in situations where large amounts of measurement error are present that result in biases that counteract one another (and with varying degrees over time). Nevertheless, applying both methods would be useful to help contextualize our findings. If both methods are in general agreement, as they were in our illustration, it would give the researcher more confidence in the magnitude of measurement error present and the potential for that error to significantly affect study results. If both methods are in general *disagreement* (i.e., they produce median PFS estimates that are shifted in opposite directions), this may underscore a large presence of measurement error and warrant further investigations and QBA.

Probabilistic QBA, in general, allows for appropriate propagation of error beyond the sampling error estimated in many standard statistical software packages, often resulting in wider uncertainty estimates and confidence intervals. This is to be expected as demonstrated in our example. While increased uncertainty may affect qualitative interpretations of findings, it is important to obtain estimates that reflect additional error, allowing us to make informed decisions regarding PFS even if the conclusion comes with less certainty. In some cases, estimates of uncertainty obtained via QBA may be so large that inferences drawn are inconclusive or compromised. Such results may still be informative when conducting future studies, underscoring the need to identify, quantify, and address sources of bias at the early design and analysis planning stages.

While some existing sensitivity analyses could explore alternate ways of defining the TTE outcome (e.g., considering treatment switches as PFS “events”), they can still fail to address how measurement error bias in the endpoint influences study findings. The QBA methods presented in this paper can help address such issues attributed to differences in how PFS is assessed in trial vs real-world settings when conducting ECAs. Additional applications of these methods could consider increasing the magnitude of the error parameters and estimating the survival curves using CICC and SRC under a wider range of plausible conditions. This would function like a tipping point analysis, highlighting how much measurement error would be necessary to cause substantial bias in our RWD sample.

Both CICC and SRC focus on *post hoc* shifting the survival curve rather than attempting to identify and adjust for measurement error in the initial PFS estimation process of these models. Extensions and adaptations of additional approaches, like the simulation-based approach taken by Gray et al.,^20^ may be of interest when researchers want to evaluate robustness of other quantities, like hazard ratios, to measurement error. Furthermore, while surveillance bias appears to have minimal impact in the illustrated NDMM setting, future work should also focus on methods to address when measurement error bias is attributed more to differences in assessment timing between RWD and trials.^21^ Finally, complementary methods may be further refined for other endpoints, such as binary ones like overall response rate and minimal residual disease. Further work is needed to evaluate these methods in other settings including across cancers and across other types of endpoints (e.g., imaging-based, patient reported, and more).

This work provides an example of how one can leverage knowledge from clinical experts in a simulation framework to implement QBA methods to quantify measurement error due to differences between how real-world and trial PFS is assessed. While it would be ideal for researchers to design RWD studies that collect validation data on endpoints, QBA approaches can provide valuable evidence when such designs are not possible. These methods are important for the broader scientific community that leverages RWD for regulatory and clinical decision-making when interventional studies are not feasible, allowing researchers to evaluate the impact of known sources of bias to aid in the interpretation of study findings in regulatory submissions.^2^

## Supplementary Material

Web_Material_kwag027

## Data Availability

Data available on request from the authors.
