# Supplementary material for: Quantitative bias analyses to address measurement error in time-to-event endpoints
Source: Am J Epidemiol. 2026 Feb 6;195(9):2476–83. doi: 10.1093/aje/kwag027 (PMC13537843; doi:10.1093/aje/kwag027)
Supplement: Supplementary file 1 [file web_material_kwag027.zip › AJE-01252-2025_supplement_ACCEPTED_CLEAN.docx]

**Supplementary Data for “Quantitative bias analyses to address measurement error in time-to-event endpoints”**

**AUTHORS:** Benjamin Ackerman, Ryan W. Gan, Youyi Zhang, Jennifer Hayden, Jocelyn R. Wang, Craig S. Meyer, Juned Siddique, Jennifer L. Lund, Janick Weberpals, Sebastian Schneeweiss, Til Stürmer, James Roose, Omar Nadeem, Noopur Raje, Sikander Ailawadhi, Smith Giri, Laura Hester, Jason Brayer, Ashita S. Batavia

**Included Materials:**

- Table S1: Abbreviations
- Figure S1: Time between assessments according to trial-like assessment schedule vs. real-world cadence. Time between visits has greater variability in routine clinical practice than in clinical trials.
- Appendix S1: Example Simulation and QBA application code

**Table S1:** Abbreviations

| CICC | Cumulative incidence curve correction |
| --- | --- |
| ECA | External control arm |
| FDA | Food and Drug Administration |
| FHRD | Flatiron Health Research Database |
| FLC | Free-light chains |
| IMWG | International Myeloma Working Group |
| mPFS | Median progression-free survival |
| NDMM | Newly diagnosed multiple myeloma |
| PFS | Progression-free survival |
| QBA | Quantitative bias analysis |
| RWD | Real-world data |
| RWE | Real-world evidence |
| rwPFS | Real-world progression-free survival |
| SPEP | Serum protein electrophoresis |
| SRC | Survival regression calibration |
| TTE | Time-to-event |
| UPEP | Urine M-protein |

**Figure S1:** Time between assessments according to trial-like assessment schedule vs. real-world cadence. Time between visits has greater variability in routine clinical practice than in clinical trials.

**Appendix S1: Example Simulation and QBA application code**

Additional simulation methods details can be found here: Ackerman, B., Gan, R. W., Meyer, C. S., Wang, J. R., Zhang, Y., Hayden, J., et al. (2024). Measurement error and bias in real-world oncology endpoints when constructing external control arms. Frontiers in Drug Safety and Regulation, 4, 1423493.

```
library(tidyverse)
library(ggsurvfit)

# Specify parameters ----
# “True” data generating mechanism
N_SIM <- 1000 # Number of simulation iterations to conduct
N <- 365 # Sample size of study for simulation
MEDIAN_TIME <- 1026 # Median time to event (in days)
END_OF_STUDY <- 1686 # End of study duration (in days)
TRIAL_CYCLE <- 28 # Treatment cycle duration of single-arm trial

# “Measurement error” data generating parameters
# Misclassified events
SENSITIVITY <- 0.5 # Probability that event status is missed/delayed
SPECIFICITY <- 0.8 # Probability that event status is earlier than detected per trial standard
# Assessment cadence
PROB_IRREGULAR_ASSESSMENT <- 0.7 # Probability that RWD assessments vary from trial schedule
SD_IRREGULAR_ASSESSMENTS <- 0.9 # Variability of RWD schedule from trial schedule

QBA_parameters <- tibble() # Initiate table for storing QBA parameters

for(i in 1:N_SIM){

  # Simulate true event times and align to a trial cadence ----
  true_time <- sort(rexp(N, rate = log(2) / MEDIAN_TIME))
  scheduled_time <- ceiling(true_time / TRIAL_CYCLE) * TRIAL_CYCLE
  event <- as.numeric(scheduled_time < END_OF_STUDY)

  # Simulate event times under misclassification bias ----
  false_negatives <- rbinom(sum(event), 1, prob = 1 - SENSITIVITY)
  false_positives <- rbinom(N - sum(event), 1, prob = 1 - SPECIFICITY)

  misclassified <- c(false_negatives, false_positives)

  time_bias <- rexp(N, rate = log(2) / EVENT_DETECTION_TIME_BIAS)
  mismeasured_time <- ifelse(misclassified == 1,
                             scheduled_time + c(false_negatives, false_positives * -1) * time_bias,
                             scheduled_time)

  # Adhere to trial-like cadence
  mismeasured_scheduled_time <- ceiling(mismeasured_time / TRIAL_CYCLE) * TRIAL_CYCLE

  # Simulate event times under misclassification bias + surveillance bias ----
  cadence_mixture_component <- sample(1:2, size = N, replace = T, prob = c((1 - PROB_IRREGULAR_ASSESSMENT), PROB_IRREGULAR_ASSESSMENT))

  irregular_cadence_event_time <- 1:N %>%
    map(~ceiling(
      rlnorm(
        n = 1,
        meanlog = log(mismeasured_scheduled_time[.x]), # Event time is centered around what's observed
        sdlog = c(0.02, SD_IRREGULAR_ASSESSMENTS)[cadence_mixture_component[.x]] # SD generates variability around cadence
      )
    )) %>%
    reduce(c)

  # Put together simulated data ----
  analysis_df <- tibble(
    true_time = pmin(scheduled_time, END_OF_STUDY),
    true_event = event,
    mis_time = pmin(irregular_cadence_event_time, END_OF_STUDY),
    mis_event = as.numeric(irregular_cadence_event_time < END_OF_STUDY)
  )

  # Translate simulated 'validation sample' into QBA parameters ----
  # Survival Regression Calibration method
  weib_true <- survreg(Surv(true_time, true_event) ~ 1, dist = "weibull", data = analysis_df)
  weib_true_int <- weib_true$coefficients[[1]]
  weib_true_shape <- 1/weib_true$scale

  weib_obs <- survreg(Surv(mis_time, mis_event) ~ 1, dist = "weibull", data = analysis_df)
  weib_obs_int <- weib_obs$coefficients[[1]]
  weib_obs_shape <- 1/weib_obs$scale

  bias_int <- weib_true_int - weib_obs_int
  bias_shape <- weib_true_shape - weib_obs_shape

  # Cumulative Incidence Curve Correction method
  valdat <- analysis_df %>%
    mutate(tp = as.numeric(mis_event == 1 & true_event == 1),
           fp = ifelse((true_event == 0  & mis_event == 1) |
                         (mis_time < true_time & true_event == 1 & mis_event == 1), 1, 0),
           pt_fp = pmin(true_time, mis_time),
           pt_tp = pmax(true_time, mis_time) - true_time)

  est_theta <- sum(valdat$tp == 1 & valdat$true_time == valdat$mis_time)/sum(valdat$true_event)
  est_fp_rate <- sum(valdat$fp)/sum(valdat$pt_fp)
  est_tp_rate <- sum(valdat$tp[which(valdat$pt_tp > 0)])/sum(valdat$pt_tp)
  # Fix rate to be 1 when sensitivity is 1
  est_tp_rate = ifelse(is.infinite(est_tp_rate) | is.nan(est_tp_rate), 1, est_tp_rate)

  # Save all QBA parameters for this dataset
  QBA_parameters <- bind_rows(
    QBA_parameters,
    tibble(
      src_bias_int = bias_int,
      src_bias_shape = bias_shape,
      cicc_theta = est_theta,
      cicc_lambda_fp = est_fp_rate,
      cicc_lambda_d = est_tp_rate
  )
  )

}

# Apply QBA methods to real-world dataset ----
# To demonstrate how the code works ONLY, we use the 'myeloma' data from the 'survival' R package. Please do not interpret this as a substantive application or finding. Change variable names as needed for your application.

rwd <- survival::myeloma

# Get unadjusted survival estimate
surv_unadj_nonboot <- survfit(Surv(futime, death) ~ 1, data = rwd) %>%
  tidy() %>%
  mutate(type = "Unadjusted RWD",
         Ft = 1 - estimate) %>%
  rename(surv = estimate)

# Bootstrap resample patients to account for sampling variability
rwd_bootstrapped <- 1:N_SIM %>%
  map(~rwd %>%
        slice_sample(prop = 1, replace = T)
  )

# Fit models to unadjusted data
unadj_weibull <- 1:N_SIM %>%
  map(
    ~survreg(Surv(futime, death) ~ 1, dist = "weibull", data = rwd_bootstrapped[[.x]])
  )

unadj_surv <- 1:N_SIM %>%
  map(
    ~survfit(Surv(futime, death) ~ 1, data = rwd_bootstrapped[[.x]]) %>%
      tidy_survfit(times = surv_unadj_nonboot$time) %>%
      mutate(type = "Unadjusted RWD",
             Ft = 1 - estimate) %>%
      rename(surv = estimate)
  )


### Adjust with CICC ###
cicc_qba <- 1:N_SIM %>%
  map(~surv_unadj[[.x]] %>%
        mutate(time = time / 30) %>%
        rowwise() %>%
        mutate(
          bt_approx = (1 - exp(-QBA_parameters$cicc_lambda_fp[[.x]] * time)),
          # UPDATED BASED ON LATEST EDWARDS PAPER DRAFT:
          yt = ifelse(QBA_parameters$cicc_lambda_fp[[.x]] == 0, 0, (1 - exp(-QBA_parameters$cicc_lambda_fp[[.x]] * (time / 2)))),
          xt = (QBA_parameters$cicc_theta[[.x]] * (1 - yt)),
          zt = (1 - exp(-QBA_parameters$cicc_lambda_d[[.x]] * (time / 2))) * (1 - (yt + xt)),
          at_approx = xt + yt + zt,
          at_approx = ifelse(at_approx == 0 & time == 0, 1, at_approx)
        ) %>%
        ungroup() %>%
        mutate(
          # Ft_corr = cummax((Ft_obs - b)/(a - b)),
          Ft_corr_approx =  cummax((Ft - bt_approx)/(at_approx - bt_approx)),
          # Need to cap corrected cumulative incidence at 1...
          Ft_corr_approx = if_else(Ft_corr_approx > 1, 1, Ft_corr_approx),
          pfs_corrected_approx = 1 - Ft_corr_approx)
  ) %>%
  reduce(bind_rows)

cicc_qba_avg <- cicc_qba %>%
  group_by(time) %>%
  summarise(surv_mean = mean(pfs_corrected_approx, na.rm = T),
            surv_2.5 = quantile(pfs_corrected_approx, prob = 0.025, na.rm = T),
            surv_97.5 = quantile(pfs_corrected_approx, prob = 0.975, na.rm = T)) %>%
  mutate_at(vars(contains("surv")), cummin)

# Calculate mPFS and 95% CI
cicc_qba_avg %>%
  filter(surv_mean <= 0.5) %>%
  filter(surv_mean == max(surv_mean)) %>%
  slice(1) %>%
  pull(time)

cicc_qba_avg %>%
  filter(surv_2.5 <= 0.5) %>%
  filter(surv_2.5 == max(surv_2.5)) %>%
  slice(1) %>%
  pull(time)

cicc_qba_avg %>%
  filter(surv_97.5 <= 0.5) %>%
  filter(surv_97.5 == max(surv_97.5)) %>%
  slice(1) %>%
  pull(time)

### Adjust with SRC ###
# Helper function to estimate survival probabilities over time after calibrating parameters
weib_surv_curv <- function(
    linpred, scale, intercept_bias = 0, shape_bias = 0,
    time_vector = seq(0,80, by = 0.1)
) {

  # Calculate shape based on linear predictors provided
  shape = exp( sum(linpred) + intercept_bias )
  scale_adj = scale + shape_bias
  # Calculate survival probabilities
  surv = exp( -( time_vector / shape )**scale_adj )

  # Create a data frame with time and survival probabilities
  res = data.frame( time = time_vector, surv = surv )

  return( res  )

}

src_qba <- 1:N_SIM %>%
  map(~weib_surv_curv(linpred = unadj_weibull[[.x]]$coefficients,
                      scale = 1/unadj_weibull[[.x]]$scale,
                      intercept_bias = QBA_parameters$src_bias_int[[.x]],
                      shape_bias = QBA_parameters$src_bias_shape[[.x]],
                      time_vector = unadj_surv[[.x]]$time)) %>%
  reduce(bind_rows)

src_qba_avg <- src_qba %>%
  group_by(time) %>%
  summarise(surv_mean = mean(surv),
            surv_2.5 = quantile(surv, prob = 0.025),
            surv_97.5 = quantile(surv, prob = 0.975))

# Calculate mPFS and 95% CI
src_qba_avg %>%
  filter(surv_mean <= 0.5) %>%
  filter(surv_mean == max(surv_mean)) %>%
  pull(time)/30

src_qba_avg %>%
  filter(surv_2.5 <= 0.5) %>%
  filter(surv_2.5 == max(surv_2.5)) %>%
  pull(time)/30

src_qba_avg %>%
  filter(surv_97.5 <= 0.5) %>%
  filter(surv_97.5 == max(surv_97.5)) %>%
  pull(time)/30


### Plot findings all together ##
qba_example <- ggplot() +
  geom_hline(aes(yintercept = 0.5), lty = 2, col = "grey") +
  geom_line(data = surv_unadj_nonboot, aes(x = time/30, y = surv), col = "black") +
  geom_ribbon(data = surv_unadj_nonboot, aes(x = time/30, ymin = conf.low, ymax = conf.high), fill = "black", alpha = 0.1) +
  geom_line(data = src_qba_avg, aes(x = time/30, y = surv_mean), col = "darkorange") +
  geom_ribbon(data = src_qba_avg, aes(x = time/30, ymin = surv_2.5, ymax = surv_97.5), fill = "darkorange", alpha = 0.1) +
  geom_line(data = cicc_qba_avg, aes(x = time, y = surv_mean), col = "royalblue") +
  geom_ribbon(data = cicc_qba_avg, aes(x = time, ymin = surv_2.5, ymax = surv_97.5), fill = "royalblue", alpha = 0.1) +
  labs(x = "Time (months)", y = "PFS Probability",
       title = "Progression-free survival by method",
       subtitle = "Unadjusted (black), SRC QBA (orange), CICC QBA (blue)") +
  theme_minimal()
```
